# Expanded Transposition Flap Technique for Total and Subtotal Resurfacing of the Face and Neck

**Published:** 2007-04-30

**Authors:** Robert J. Spence

**Affiliations:** Johns Hopkins School of Medicine, Baltimore, MD

## Abstract

**Background:** The reconstruction of major burn and other deformities resulting from significant soft tissue deficits of the face and neck is a continuing challenge for surgeons who wish to reliably restore facial function and aesthetic appearance. A primary problem is deficiency of well-matched donor skin. Other problems include the unique characteristics of facial skin, the fine anatomic nuances, and the unique functional demands placed on the face. This article describes an expanded shoulder transposition flap that can provide a large amount of both flap and full-thickness skin graft for total and subtotal reconstruction of the face. **Methods:** An expanded shoulder transposition flap has been used since 1986 for head and neck resurfacing 58 times in 41 patients ranging in age from 2 to 62 years. The details of the technique and the results of the flap including complications are described. **Results:** The flap proved remarkably reliable and reproducible in resurfacing the peripheral facial aesthetic units. The pedicle skin is often used for grafting of the central face with its finer features. The donor site of the flap is closed primarily. **Conclusions:** Twenty years' experience with expanded transposition flaps has shown it to be reliable and versatile in the reconstruction of major soft tissue deficits of the face and neck. It is a technique that provides economy of tissue, versatility, and is well within the skill, patience, and courage of most reconstructive surgeons.

The reconstruction of major burn and other deformities resulting from significant soft tissue deficits of the face and neck is a continuing challenge for surgeons who wish to reliably restore facial function and aesthetic appearance. The face is very difficult to reconstruct. The primary problem in burn deformities is a frequent deficiency of well-matched donor skin. On a finer and more complicated level, the unique characteristics of facial skin and the unique functional demands placed on the face make it difficult to reconstruct.

Many techniques exist for facial resurfacing including, most recently, allotransplantation. Each technique has its advantages, but most have significant problems resulting in less than optimal results. The best results reported have often been isolated cases from surgeons of great skill, patience, and courage.

Cherry^1^ and Sasaki^2^ independently described extended survival length with expanded random flaps, and described the rich vascularity of the capsules of tissue-expanded skin. Based on that work, we first began using tissue expanded skin as transposition flaps in 1986 to provide thin, reliable coverage of large areas of the face and neck.^3^ The expanded skin derived from the well-matched local skin of the shoulders and upper torso provides increased area of skin for transfer, and allows primary donor site closure.

In the 20 years we have used this flap, we have found it to be remarkably reliable and well within the skill, patience, and courage of most reconstructive surgeons. This article describes this technique and reviews the pertinent details of this 20-year experience.

## METHODS

The records and photographs of all our patients treated with one or more tissue-expanded transposition flaps (XTFlaps) for deformities of the face and neck were reviewed. Forty-one patients were treated with 58 XTFlaps over 20 years. The patients ranged in age from 2 to 62 years. Eight were children, 5 male and 3 female, in whom 12 XTFlap procedures were performed. Of the 33 adults, there were 22 males and 11 females (Table 1).

### Preoperative preparation

Before operating, the donor site for the XTFlap is determined by evaluating those areas close to the face and neck with unscarred, well-matched skin. Most often the donor site is the shoulder. The area available for a tissue expander pocket is measured and an appropriate-sized rectangular tissue expander is obtained. Most commonly, adult men require an approximately 15 × 8-cm tissue expander. In women, a 13 × 7-cm tissue expander is common.

### Tissue expander placement

After undergoing appropriate preoperative evaluation, the patient is placed under general endotracheal anesthesia. An intravenous antibiotic (usually a first-generation cephalosporin) is given. A roll is placed under the shoulder if this is the donor site, so that dissection can be carried somewhat posterior to the superior border of the trapezius muscle.

The area of the proposed tissue expander pocket is marked using the dimensions of the proposed tissue expander. Most often an incision approximately 4 cm long is made midway along and parallel to the anterior margin of the proposed tissue expander pocket. The orientation of this incision is important as it will be included in the incision when the transposition flap is raised after expansion. For shoulder tissue expanders, this incision frequently lies along the middle of the clavicle. If a scar is present in the area, every effort is made to use it for access to the tissue expander site.

The incision is made to the muscle fascia, and the skin and subcutaneous tissue are undermined using the coagulation cautery on the fascia. The undermining is carried out to the extent of the previously marked dimensions. After irrigating with saline, obtaining meticulous hemostasis, and irrigating again with half povidone iodine and half saline solution, the tissue expander is placed. When an integral valve is used, 2 or 3 absorbable sutures are placed fixing either the port or the base plate (depending on the type of expander) so that the tissue expander cannot roll over. The apron around the port is preferred, as sutures here also prevent folding of the deflated tissue expander wall interposing itself between skin and port. The wound is then closed with absorbable deep sutures: interrupted simple sutures in the superficial fascia and interrupted inverted dermal sutures. Skin tape or tissue adhesive is applied for final skin approximation after the tissue expander is inflated to the point of mild tension on the overlying skin. I prefer not to have any sutures through the epidermis to minimize the possibility of bacterial entry into the tissue expander cavity. No drain is placed unless an unusual amount of bleeding is seen or anticipated. The patient is usually discharged the same day.

### Tissue expander inflation

**Figure 1:**
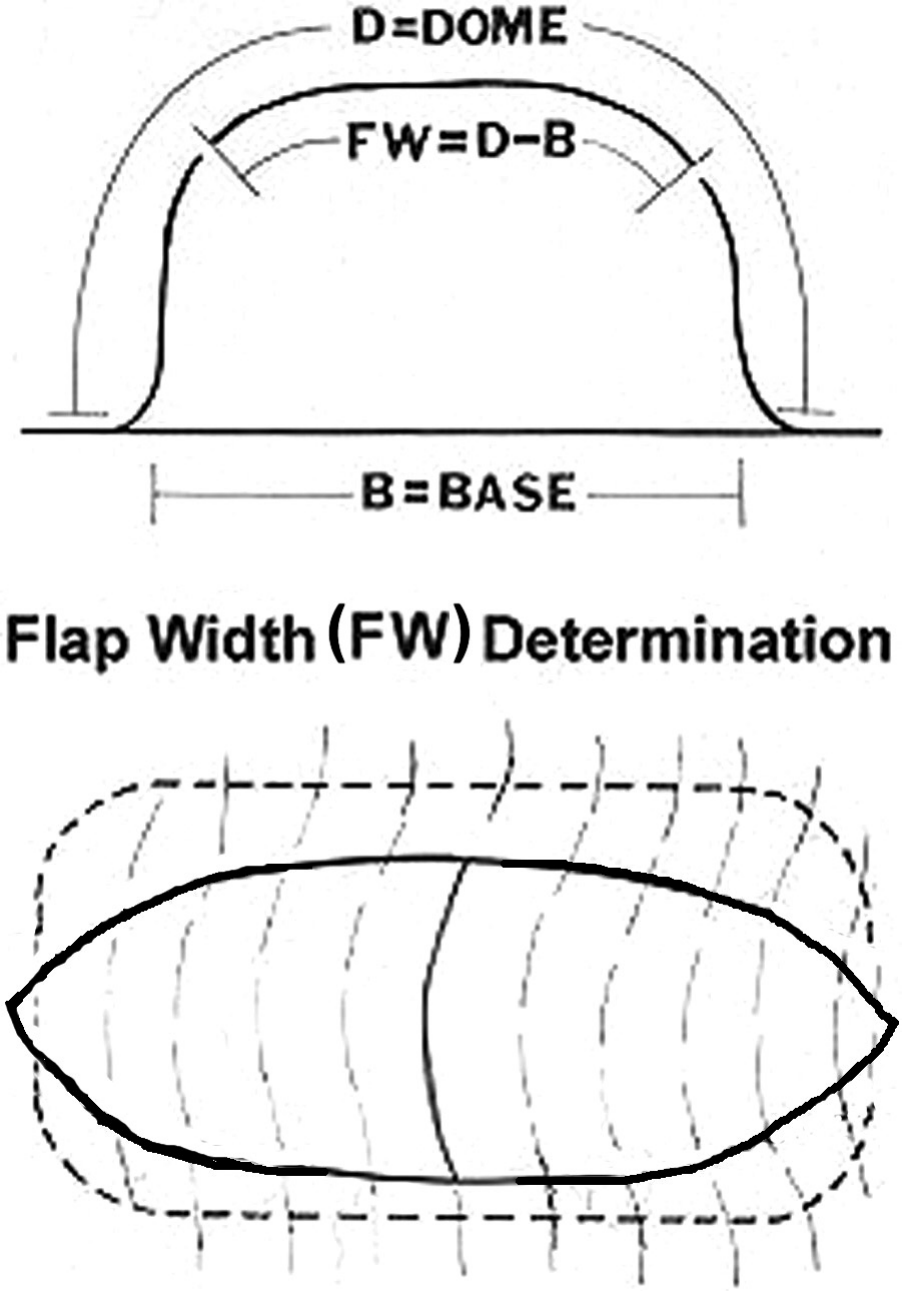
The amount of available expanded skin for the width of the flap is measured by subtracting the distance across the base of the tissue expander from the distance measured over the dome. This leaves enough expanded skin to close the donor site once the flap has been transposed.

Tissue expander inflation begins 2 weeks after insertion of the expander. Expanders are inflated to patient tolerance weekly. Measurements of the base and the distance over the dome of the expander are used to determine the amount of new skin available for the transposition flap. The base width is subtracted from the distance over the dome to determine the potential width of the transposition flap (Fig 1).

The length of the flap is measured longitudinally over the dome. A wide lenticular-shaped pattern can then be constructed and compared to the normal contralateral facial unit to be resurfaced or to the proposed defect on the affected side. When more than enough skin is available, inflation is stopped and a flap transposition is performed 2 weeks after final injection. The average length of tissue expansion is 3 months measured from the time of initial insertion of the tissue expander to transposition of the flap.

### Transposition of the flap

**Figure 2:**
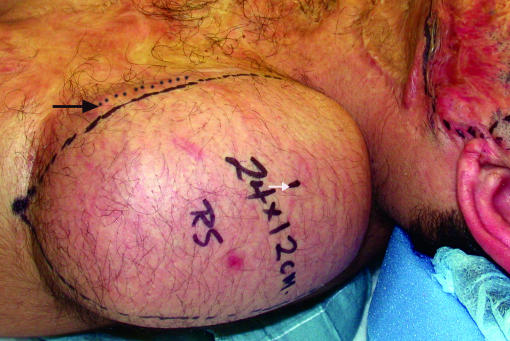
A widened, V-shaped pattern of available skin is marked on the lateral two-thirds of the expanded skin, with the scar from the insertion of the tissue expander as the anterior margin of the marking (black arrow). The long axis of the pattern is marked at half the distance between anterior and posterior margins (white arrow).

Again under general anesthesia, the recipient site on the face or neck, the expanded flap donor site, and a split-thickness skin graft donor site on the thigh are prepared. A final measurement of the amount of skin available is made as described above (Fig 1), and a widened, V-shaped pattern of available skin is marked on the lateral two-thirds of the expanded skin *with the scar from the insertion of the tissue expander as the anterior margin of the marking*. This initial V-shaped pattern, which is the lateral 60% of the lenticular-shaped piece of available expanded skin, will be slightly modified and/or reoriented on the basis of the pattern from the defect made by the scar excision or contracture release. The long axis of the pattern is marked at half the distance between anterior and posterior margins (Fig 2). This is helpful in locating the lateral tip of the flap, which is initially marked at the lateral end of the long axis just off the tip of the tissue expander.

**Figure 3:**
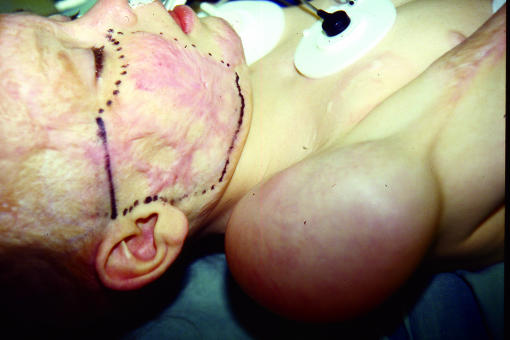
The cheek aesthetic unit is outlined leaving all available eyelid skin.

The aesthetic unit to be covered with the flap is first addressed by marking the proposed incisions and injecting a dilute solution containing epinephrine along the line just deep to the dermis. As much normal eyelid skin as possible is preserved by marking along the U-shaped eyelid-cheek junction (Fig 3).

**Figure 4:**
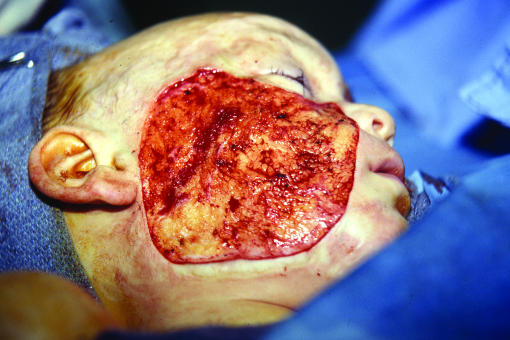
In this early case, initial excision of the cheek aesthetic unit excluded the triangles anterior to the tragus and at the anterior mandible marionette line junction.

Frequently, the triangle anterior to the tragus and the triangle where the marionette line meets the mandibular margin are left intact at the initial incision (Fig 4). The apices of these 2 triangles represent the greatest width of the cheek aesthetic unit, and they can be removed when adequate flap is ensured after transposition and partial inset later in the operation.

Any small flap that might be useful for transposition into an adjacent aesthetic unit is designed and executed before excising the rest of the scarred aesthetic unit. For example, a nasolabial flap for reconstruction of the nasal ala is commonly raised and inset before excising the rest of the cheek aesthetic unit.

The aesthetic unit is excised just deep to the dermis with the electrocautery. Once excised, a template of the resulting wound is made, and the wound is covered with a moist pad. *Along the U-shaped margin of the lower eyelid, the template is marked as a straight line from the lateral canthus to the medial canthus*. This provides extra flap tissue to this area preventing an ectropion as the flap later migrates inferiorly from contraction and in response to gravity.

**Figure 5:**
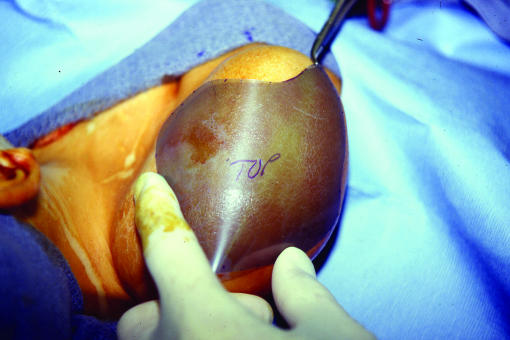
A pattern of the cheek wound is used to check the size and to orient and, in some cases, modify the shape of the previously marked V-shaped pattern on the expanded skin. The most distal point of the wound marked on the template is carefully noted and placed most laterally on the expanded skin.

The template is used to check the size, orient, and, in some cases, modify the shape of the previously marked V-shaped pattern on the expanded skin. The most distal point of the wound is marked on the template (Fig 5). Typically, this is the portion of a cheek defect lying at the junction of the nose and the medial canthus. That point on the template is placed at the previously marked point of the V-shaped pattern on the expanded skin. The template is oriented so that the narrowest dimension is directed anteriorly and posteriorly.

If the expansion has provided ample expanded skin, the template should fit within the marked pattern leaving as much as an extra centimeter of expanded skin in many areas. In aligning the template, the original lenticular pattern may have to be slightly reoriented or expanded to incorporate a slightly larger area of the template. Any lenticular-pattern skin excess is incorporated into the flap by cutting on the pattern wider than the template. This will reduce the tension when the flap is inset, and ample skin will provide the best result.

Note that the pattern is only used to help in the final orientation and modification of the original V-shaped pattern drawn on the expanded skin. The lateral portion of the flap is always incised and raised as that half of the lenticular pattern. The pattern from the defect is simply to help with orientation and slight modifications of the flap pattern. One does not incise on a line drawn from the pattern except in the case where it coincides with the lenticular flap markings. The flap is very pliable, and, after initially contracting considerably when raised, has a great capacity to stretch and reorient into the various areas of the facial or neck defect where it is required.

Final marking of the medial third of the expanded skin is delayed until the flap is elevated to the point where the larger blood vessels extending into the base of the flap within the tissue expander capsule can be determined by transillumination. The actual incisions will be modified from these original lenticular lines as the pedicle is modified to preserve the vessels at the base of the flap and to allow transposition of the flap into position on the face or neck.

**Figure 6:**
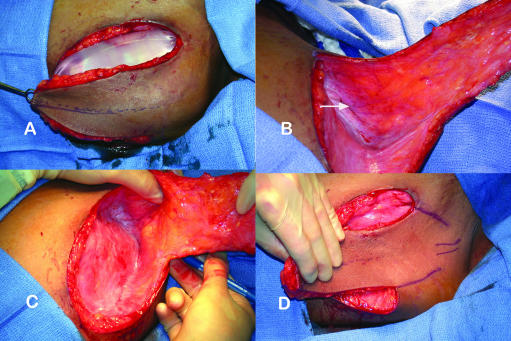
(A) The lateral 60% of the flap is raised off the tissue expander. (B) The vessels in the medial base of the expanded skin just deep to the capsule are observed. (C) A marker is pushed just anterior and posterior to these vessels and drawn back to the respective incisions, (D) leaving markings for further incisions to allow transposition onto the defect.

The flap margins are then incised into subcutaneous tissue starting laterally along the anterior and posterior margins of the final pattern. Obviously, the scar from the insertion site at the anterior margin of the flap is excluded from the flap. Medially, the pedicle is not incised (Fig 6A). Once into subcutaneous tissue, the remaining subcutaneous tissue and tissue expander capsule are incised using the coagulation cautery. Once through the skin, this incision is beveled away from the skin paddle to include excess capsule with the flap. The capsule may be tacked to the flap with absorbable sutures distally. As the flap is raised, the tissue expander can be removed. The vessels within the medial expanded skin and just superficial to the capsule can now be seen once the lateral half of the flap has been raised (Fig 6B). This can be done especially well with transillumination. One or two of the largest vessels in close proximity to each other are selected. The pedicle is now redesigned with these vessels at the center of its base. A marker is pushed into the medial skin anterior and posterior to the vessels to be preserved, and lines of incision are marked from these points laterally to their respective incisions already made (Figs 6C and 6D). When this is done, I have found that the pedicle can be narrowed considerably as long as these vessels remain in continuity. The pedicle is then cut progressively medially and even “back-cut,” if necessary, to allow transposition of the flap into position. If a vessel has to be approached very closely to allow transposition, a hypodermic needle passed from the capsule side out through the skin is helpful to mark the absolute limit of the medial incision. However, the flap pedicle is narrowed only to the point where easy transposition is accomplished with minimal tension on the tip of the flap, and very close approach to the vessels is uncommon.

**Figure 7:**
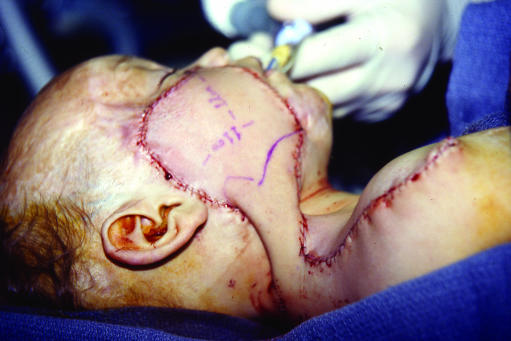
The flap is inset and the donor site is closed. The tubed pedicle is closed in a spiral fashion so that very little open wound is left.

Transposition of the flap to the cheek causes the pedicle to tube itself in a spiral fashion. Transposition of the flap to the neck forms a “dog ear” at the pedicle. The flap is inset. Dermal sutures with 3-0 absorbable sutures are placed first to orient the critical points and absorb any tension of wound closure. Two 4-0 clear nylon sutures are placed into the periosteum to hang the flap against the force of gravity: one at the lateral nose at the medial canthus to the flap tip dermis, and another at the orbital rim lateral to the lateral canthus into the flap dermis. The margin of the lower-eyelid skin is closed to the horizontal superior margin of the flap, resulting in expected redundancy there. The tubed pedicle is closed in a spiral fashion so that very little open wound is left (Fig 7).

The most proximal contact of recipient wound and flap is necessarily left unsutured. A small drain is left under the flap. Suction usually cannot be maintained owing to the open nature of the wound, so the drain acts passively. Therefore the drain should be placed as dependently as possible.

The donor site is closed over a suction drain after removing much of the remaining capsule on the shoulder, providing a new, raw wound to optimize healing. Care is taken not to remove the capsule near the pedicle base to avoid compromise of the vessels entering the flap. The flap donor site is routinely closed primarily using the residual expanded skin left there for that purpose. If preoperative measurements or estimation of the recipient wound were inaccurate, the wound may not close primarily. In that event, split-thickness skin graft from the prepared thigh donor site can be used to close the donor site. Although rarely necessary, this thigh donor site provides security that whatever is necessary for resurfacing of the defect can be obtained from the expanded donor site without regard for ultimate closure of the flap donor site.

A topical antibiotic ointment is applied to the flap inset suture line. No constricting dressing is placed near the pedicle. The donor site is dressed routinely. Postoperative orders include an admonition against anything around the neck that might compromise the pedicle. The head of the patient's bed is elevated, or else the flap will become edematous to varying degrees. Mild congestion of the flap is common. Before the operation, I discuss the possibility of leech therapy with my patients, and have leeches available. I have a low threshold for using leech therapy as added security. The patient is often discharged on the next day. Occasionally, edema in the flap in bilateral cases of cheek resurfacing will cause difficulty with vision, requiring longer hospitalization. Concern for this or flap viability will prolong what is normally an overnight stay after this operation. Antibiotics are continued until the drains are removed at approximately 6 days.

### Division and insetting

Division and insetting of the flap is routinely scheduled for 14 days after initial transposition of the flap. Again, under general endotracheal anesthesia, the pedicle is divided at its base. The base wound is closed. The pedicle is unfurled and redraped over the open end of the recipient wound, frequently reopening the flap inset for a short distance to get the best redistribution of the flap tissue along the recipient wound margin. After final preparation of the recipient wound, the proximal flap margin is marked, divided, and inset.

The residual, unscarred skin from the pedicle is frequently used as full-thickness skin graft (FTSG) to resurface another aesthetic unit. The tubed pedicle is opened along the spiral scar, the skin unfurled, and defatted. Frequently, another aspect of reconstruction is also performed during the same anesthetic, particularly if some other smaller facial flap had been used from the cheek at the previous operation to reconstruct the nose, for example.

**Figure 8:**
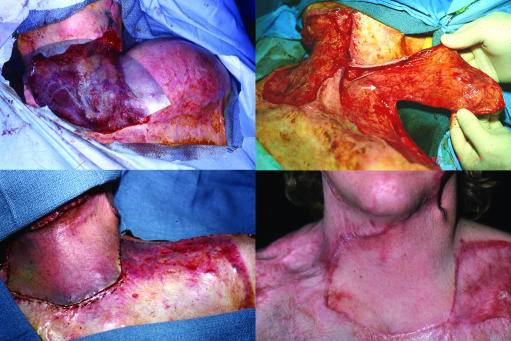
An early case of neck resurfacing. The white arrow points out the axial blood vessel medially at the base of the flap.

Figure 8 illustrates the first patient on whom I attempted an XTFlap. It shows how the flap can be transposed onto a very sizable defect of the neck. Although it provided enough skin for resurfacing, it demonstrated that having a second expanded flap from the other shoulder is optimal to ensure total resurfacing without residual cervical tightness. Expanding the skin of the contralateral shoulder also provides a second option if there is a problem with the primary tissue expander. A dog ear is frequently created when the flap is transposed. This frequently results in a secondary procedure for its excision. Occasionally, transposition of the flap onto the neck and closure of the donor site results in something similar to a Z-plasty that does not require dog ear excision.

## RESULTS

From 1986 through 2006, 41 patients were managed with 58 XTFlaps. Eight patients were children, 5 male and 3 female, in whom 12 XTFlap procedures were performed. Among the 33 adults, there were 22 males and 11 females (see Table 1).

**Figure 9:**
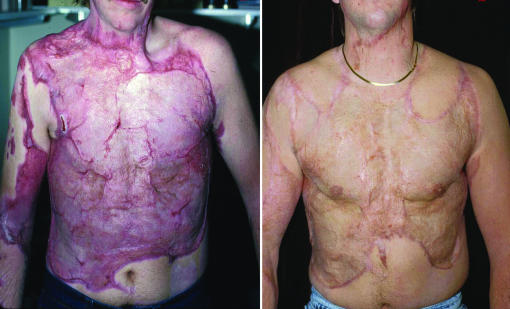
Preoperative and postoperative photos of the early neck resurfacing case after revision of the dog ear and transposition of two other back and shoulder expanded transposition flaps to resurface the scars of the shoulders.

**Figure 10:**
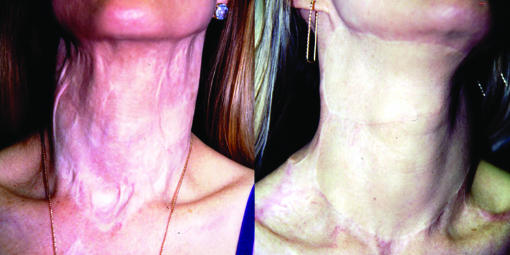
A case of total neck resurfacing of a particularly long neck with two shoulder expanded transposition flaps.

**Figure 11:**
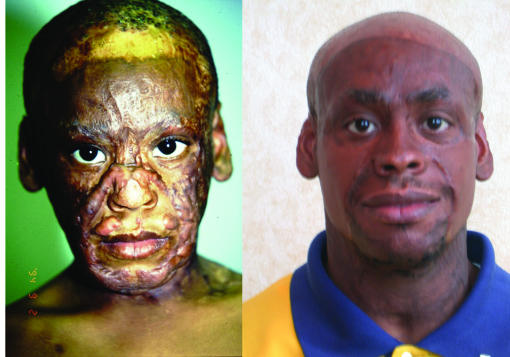
A case in which bilateral expanded transposition flaps were used to resurface the two cheek aesthetic units, and the left-sided pedicle was used as a full-thickness skin graft to resurface the patient's nose aesthetic unit.

**Figure 12:**
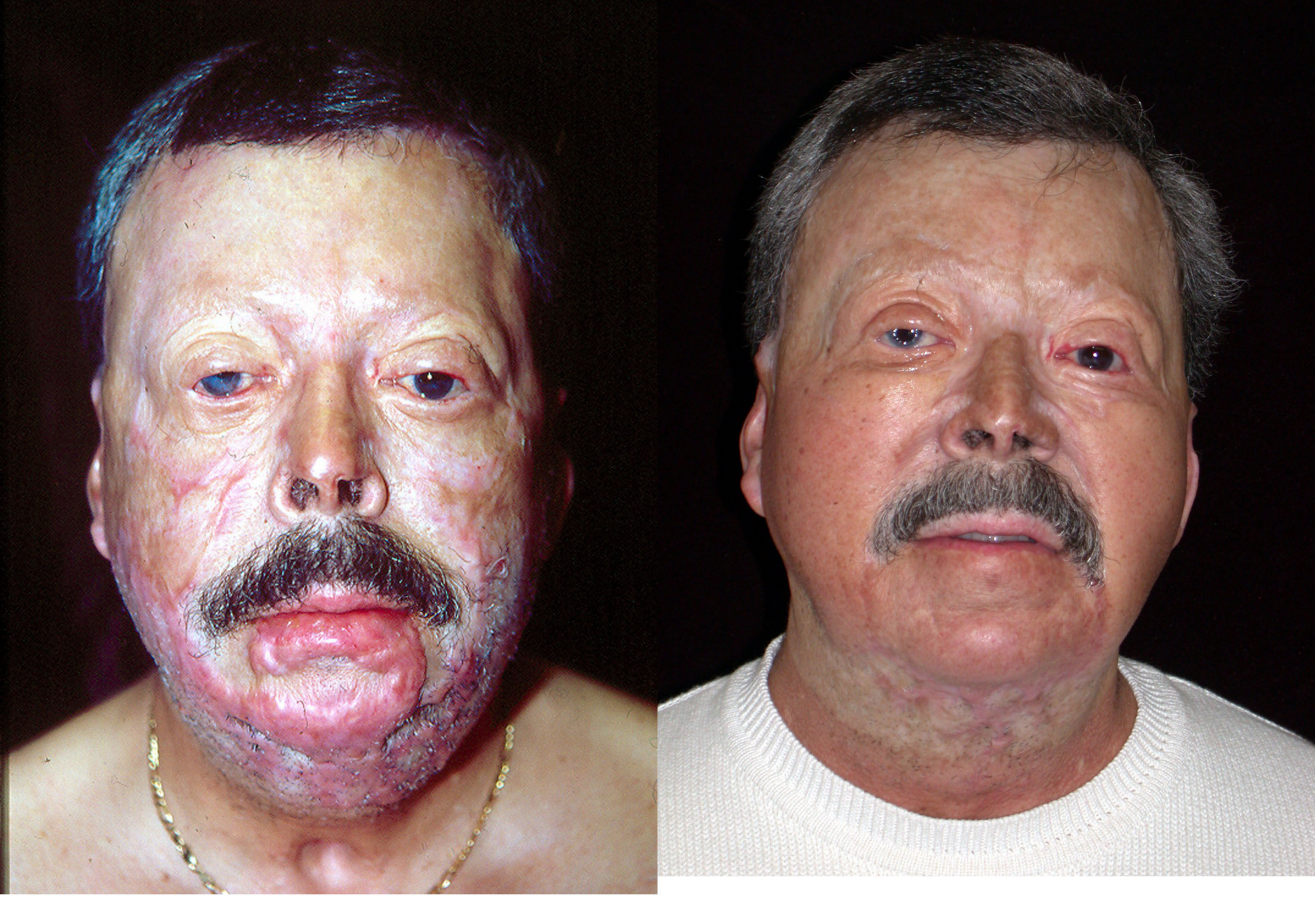
A case in which bilateral expanded transposition flaps were used to resurface both cheek aesthetic units, and the left flap pedicle was used as a full-thickness skin graft to resurface the lower lip and chin aesthetic units.

The first patient to have this flap procedure has been reported previously.^3^,^4^ This individual ultimately had 3 XTFlaps to reconstruct his neck and shoulders (Fig 9). Three other demonstrative cases are illustrated in Figures 10 to 12.

Generally, the XTFlap has proven to be exceedingly hearty and reliable. However, the 58 flaps in 41 patients resulted in some complications that were frequently instructive. These complications were generally early in the series and led to improvements in technique. Complications led to failure in transposing the flap and obtaining the desired results in only 2 cases (Table 2).

On 2 occasions, the tissue expander became exposed during the expansion process. On both occasions, the tissue expander was transposed. In one case, an attempt was made to transpose the flap, including a thin area near the exposure site on the lateral dome. This thin area of tissue subsequently died, resulting in loss of the distal quarter of the flap, the largest area of flap loss in any of my cases. In a second case, the area of exposure was simply avoided by making the flap more posterior on the expanded shoulder skin dome, providing a flap with good skin that covered the facial defect completely; this was the only time the flap donor site required a skin graft for closure.

Significant loss of a portion of a flap has occurred on 3 occasions. The largest loss was mentioned above when a thinned area of skin from around a tissue expander exposure was thought to be viable enough to transfer with the flap. On review of the records, this was the only flap that did not have a visible vessel present in the pedicle, and this was thought to be due to a scar from a skin graft donor site at the lateral base of the neck made by a previous surgeon. Another significant loss of a flap came when we attempted to transpose expanded skin that had thinning in the central portion of the expanded dome. Interestingly, only the thin skin in the central flap developed necrosis. The distal and the proximal flap both were viable. On the third occasion, necrosis of about the distal 10% to 15% of the flap required excision of the flap tip and placement of an FTSG from the pedicle.

A planned XTFlap was aborted and the flap was converted to an FTSG in only 1 case. That case, mentioned earlier, was when I attempted to transpose an expanded deltopectoral flap in 1987. I discovered that the expanded skin was too thick to allow expression of the fine features of the central face. Another XTFlap procedure of the scalp was aborted when we raised it and tried to turn it inferiorly over the mandible with the plan to replace severe hypertrophic scarring of the beard area. The flap became severely congested in the dependent position, so we replaced the flap in its donor site. The flap survived when returned to the scalp donor site, but the patient subsequently elected to abort the procedure despite our thought that the flap might do well after the delay. The excess flap skin was excised, and the scalp was closed. I had not excised the hypertrophic scar of the cheek. I subsequently have not performed any scalp XTFlaps, and my bias is that XTFlaps do better when the proximal venous drainage of the flap is dependent.

There were also the usual complications associated with all tissue expanders. An additional operation was required to replace or adjust the position of a tissue expander during the expansion process on 6 occasions. The tissue expander deflated and had to be replaced 4 times in 3 patients. One tissue expander slid forward onto the anterior chest early in the series when we tried to place the tissue expander through a radially oriented incision from the chest. One tissue expander port flipped over into a position that could not be reached by the percutaneous needle.

## DISCUSSION

Reconstructing extensive areas of facial soft tissue deformity represents one of the biggest challenges for the reconstructive surgeon. The face is of supreme importance to the patient and society, and is extremely complex anatomically, functionally, and aesthetically. When large areas of the face are abnormal and deformed, a primary reconstructive problem is the frequent lack of adequate, well-matched tissue for reconstruction.

The ideal reconstructive system is elusive. Such a system would first provide adequate well-matched skin that can be used reproducibly and reliably to return excellent function and appearance. In addition, the donor site of the tissue should be minimized and economy of tissue usage optimized to reduce further destruction of the patient's body. Of course, the ideal system would eliminate scars and deformity entirely. All of this should be within the skill and patience of most reconstructive surgeons, and, in today's world, minimize medical costs.

The XTFlap satisfies many of these requirements. In addition to providing ample well-matched skin for reconstruction of significant facial and neck soft tissue deformities, another major advantage of the XTFlap is that it appears to have the reliability of an axial flap. Clearly, the primary vessel on which I base the transposition of the flap is immediately superficial to the expander capsule. Given this, I stress the importance of *maintaining continuity of the capsule in the base of the pedicle* as the flap is being elevated, and I preserve the capsule in continuity with the pedicle on the undersurface of the XTFlap.

Basing this large flap on such a relatively narrow pedicle is the first great leap of faith associated with the procedure. Besides its reliable viability, another great advantage is the ability to close reliably both the defect and the flap donor site. This is accomplished routinely be expanding the tissue expander until measurements indicate that more than enough skin is available to achieve this. Expansion simply continues until this is achieved. The expanded skin is allowed to grow fully by stopping expansion 2 weeks before transposition of the flap. Excising an entire large aesthetic unit of the face relying on these simple measurements across the base and over the dome of the tissue expander is a second great leap of faith.

Another major advantage is the availability of the residual expanded skin that the pedicle provides after division and insetting of the flap. This “bonus” skin has proven exceedingly valuable when used as FTSG in resurfacing the central portion of the face where preservation of the fine facial features is paramount.

Taken together, the advantages of the XTFlap in resurfacing of total and subtotal defects of the face and neck are substantial. Our 20 years' experience with this flap has shown it to be reliable and versatile for the reconstruction of major soft tissue defects of the face and neck. It is a technique that provides economy of tissue and versatility and is well within the skill, patience, and courage of most reconstructive surgeons.

## Figures and Tables

**Table 1:** Patient population

	**Gender ratio (Male:Female)**	**No. of Flaps**
33 Adults	22:11	46
8 Children	5:3	12

**Table 2:** Complications (total flaps *N* = 58)

Infections	3 (5%)
Exposure of tissue expander (TE)	2 (3%)
Significant partial flap loss	3 (5%)
Deflation requiring replacement of TE	4 (7%)
Malposition of expander/port requiring repositioning of TE	2 (4%)
